# Physeal bar resection using a patient-specific guide with intramedullary endoscopic assistance for partial physeal arrest of the distal radius

**DOI:** 10.1007/s00402-018-2985-y

**Published:** 2018-06-28

**Authors:** Satoshi Miyamura, Hiroyuki Tanaka, Kunihiro Oka, Atsuo Shigi, Shingo Abe, Hideki Yoshikawa, Tsuyoshi Murase

**Affiliations:** 1 Department of Orthopedic Surgery, Graduate School of Medicine, Osaka University, 2-2 Yamada-oka, Suita, Osaka 565-0871 Japan; 2 Osaka University Healthcare Center, 17-1 Machikaneyama-cho, Toyonaka, Osaka 560-0043 Japan

**Keywords:** Distal radius, Intramedullary endoscopy, Langenskiöld procedure, Partial physeal arrest, Patient-specific guide, Physeal bar resection

## Abstract

The partial physeal arrest of the distal radius could result in progressive deformities and functional problems of the wrist. Despite being the most preferred surgical intervention, physeal bar resection (Langenskiöld procedure) is technically demanding. This manuscript aims to illustrate the technical tricks and present an illustrative case of premature physeal arrest of the distal radius managed with a novel method for the Langenskiöld procedure, involving complete removal of the bar using a patient-specific guide in combination with an intramedullary endoscopy technique that facilitated direct observation.

## Introduction

Physeal arrest of the distal radius might alter, impair, or completely stop the growth of the bone [1–3]. In particular, the partial physeal arrest which develops as a result of abnormal osseous or fibrous bridge (physeal bar) between the metaphysis and epiphysis in the local region of the growth plate can cause progressive deformities and functional problems of the wrist [1, 4–6]. Patients with partial physeal arrest of the distal radius pose a therapeutic dilemma—to prevent disruption of the bone growth, the physeal bar should be completely resected without damaging the healthy physeal cartilage. The cases in which further physeal growth is expected might benefit from physeal bar resection (PBR), also known as the Langenskiöld procedure [5, 7–11]. Although this procedure assures reestablishment of the normal bone growth, complete removal of the physeal bar to prevent recurrence of the bar formation is technically difficult.

To achieve precise resection, a unique Langenskiöld procedure using a patient-specific guide with intramedullary endoscopic assistance has been devised. This procedure involves three-dimensional (3-D) modeling of the physeal bar and creation of a surgical guide designed to target the bar on the basis of preoperative 3-D computer simulation. This article aims to describe its technical tricks and an illustrative case of premature physeal arrest of the distal radius that was successfully managed using this newly developed method.

## Surgical technique

Patients were routinely examined using computed tomography (CT) to identify the physeal bar. When the physeal damage was less than 50% of the growth plate and was expected to continue growing further for more than 2 years, a unique procedure which was identical to that of the original Langenskiöld procedure was recommended [10–13].

### Patient-specific guide

**Fig. 1:**
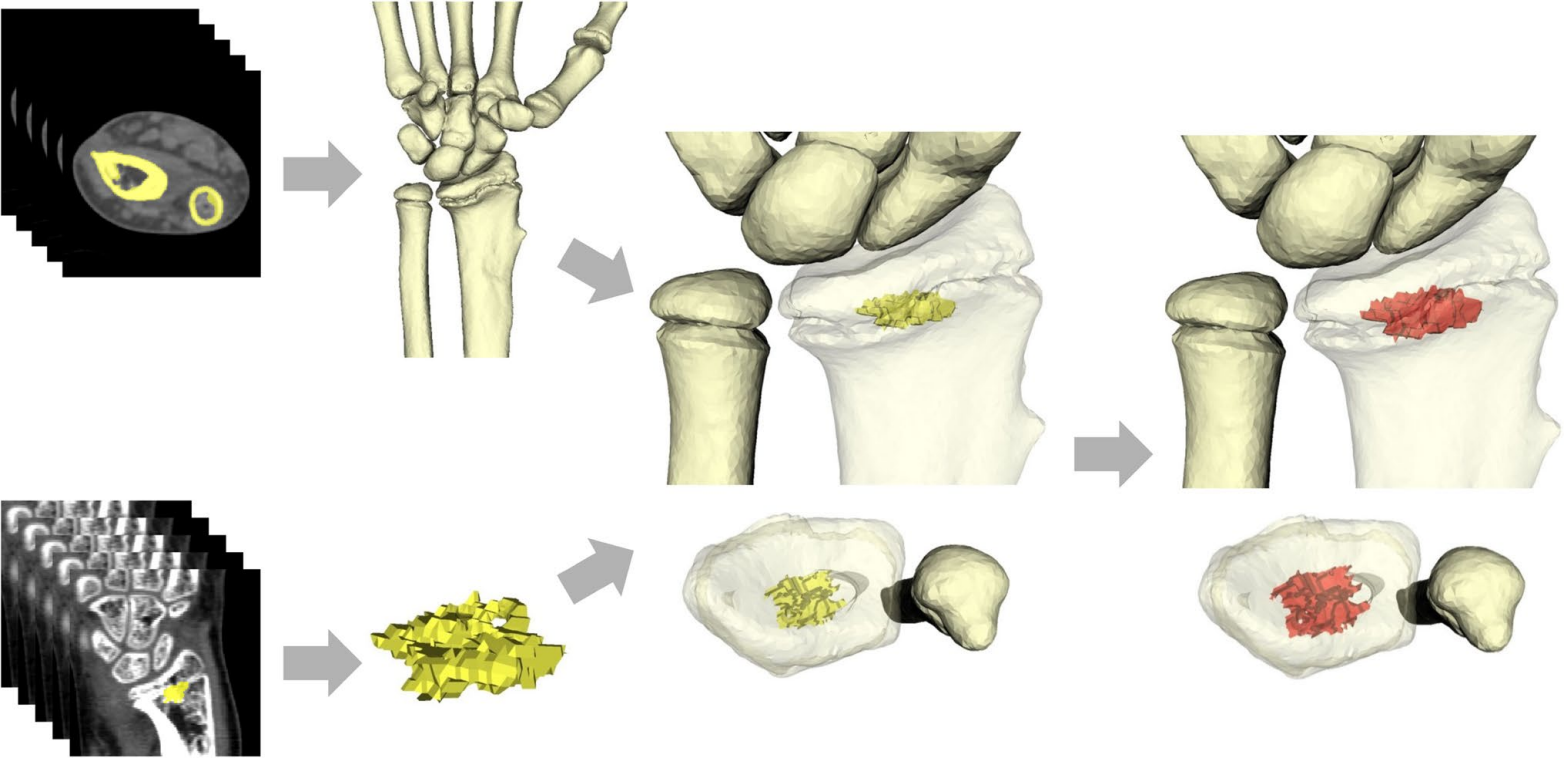
Reconstruction of 3-D models of the wrist bones and physeal bar from CT data: computer model of the physeal bar (yellow model); a target expanded the physeal bar model by 1.0 mm (red model)

**Fig. 2:**
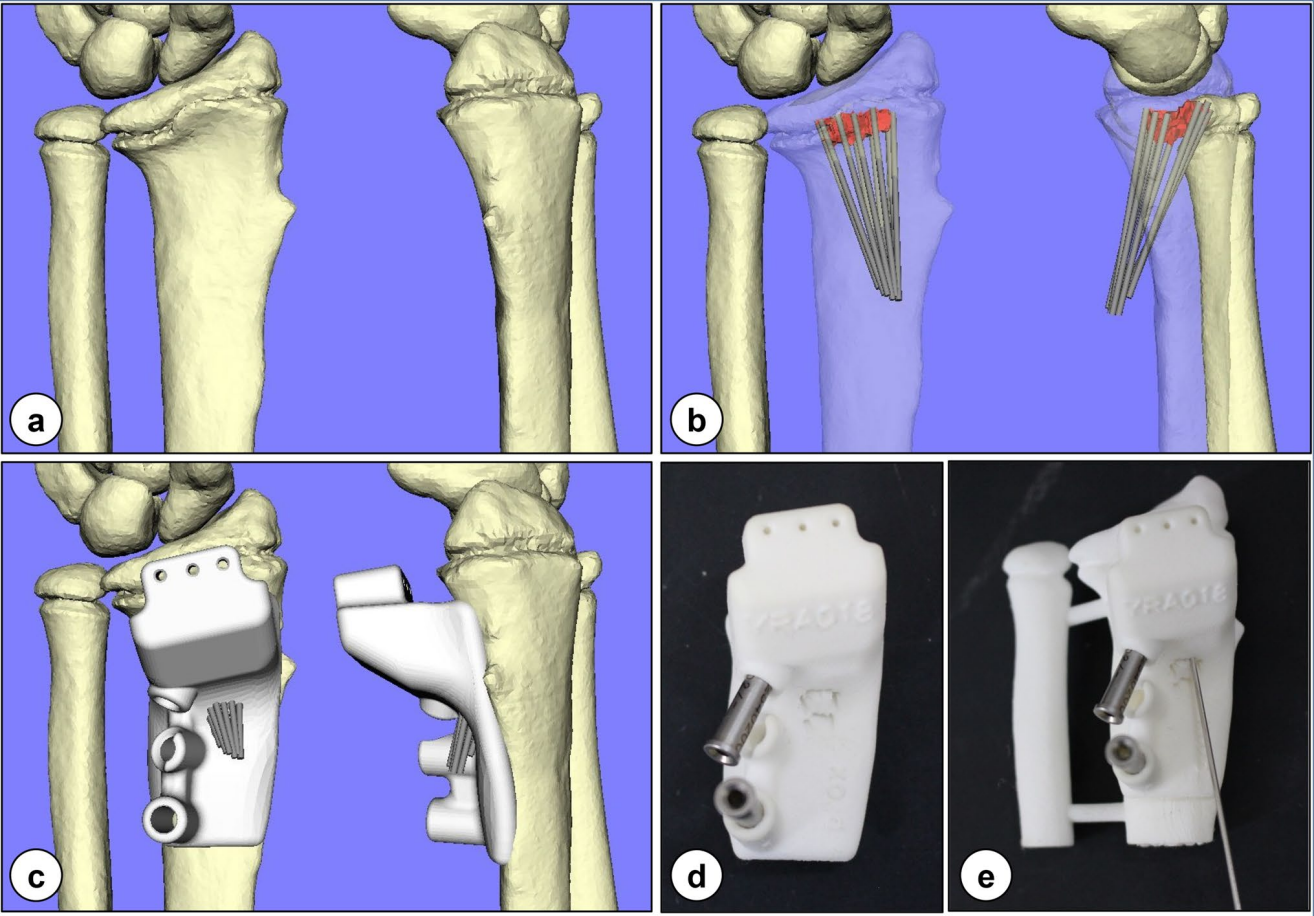
Patient-specific guide creation: **a** 3-D bone surface models; **b** 1.0-mm K-wires surrounding the target physeal bar (red) on the radius (transparent); **c** patient-specific guide with K-wires; **d** actual patient-specific guide; and **e** guide with a 1.0-mm K-wire on the radius–ulna model

The patient-specific surgical guide was created on the basis of 3-D information attained preoperatively from CT data. The 3-D surface model of the radius was created using commercially available software (Bone-Viewer™ and Bone Simulator™; Orthree, Osaka, Japan). In addition, the computer model of the physeal bar was created by manually segmenting the bridging regions connecting the epiphysis to the metaphysis to target the spatial location. The 1.0-mm expansion of the physeal bar was defined as a target to avoid both inadequate removal of the bar and excessive removal of the healthy physeal cartilage. Merging the physeal bar model onto the radius model could help attain spatial information on the physeal bar (Fig. 1). Subsequently, a patient-specific guide was created as a surgical guide in accordance with patients’ bone surface to facilitate the multiple pinning through the guide to surround the target physeal bar (Fig. 2a–e); the guide was manufactured by Teijin Nakashima Medical Co., Ltd. (Okayama, Japan) using the plastic laser sintering system [Formiga P110; Electro-Optical Systems (EOS), Munich, Germany] with a medical grade polymer (PA2200).

### Surgery

**Fig. 3:**
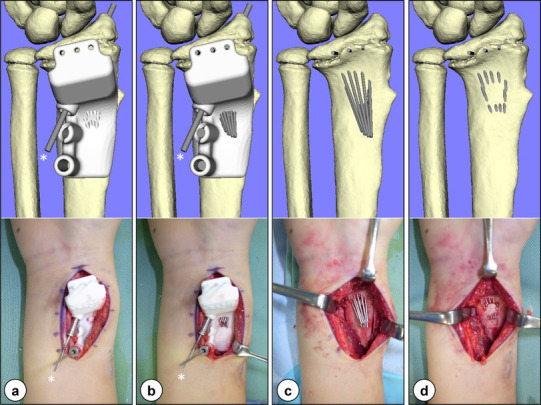
Surgical process using the patient-specific guide (upper, preoperative simulation; lower, intraoperative photograph): **a** patient-specific guide fitted onto the palmar surface of the distal radius under the guidance of the K-wire (*) designed to indicate the radial styloid tip as a reference; **b** insertion of K-wires; **c** removal of the instrument leaving the K-wires; and **d** cutting the K-wires at the bone surface

**Fig. 4:**
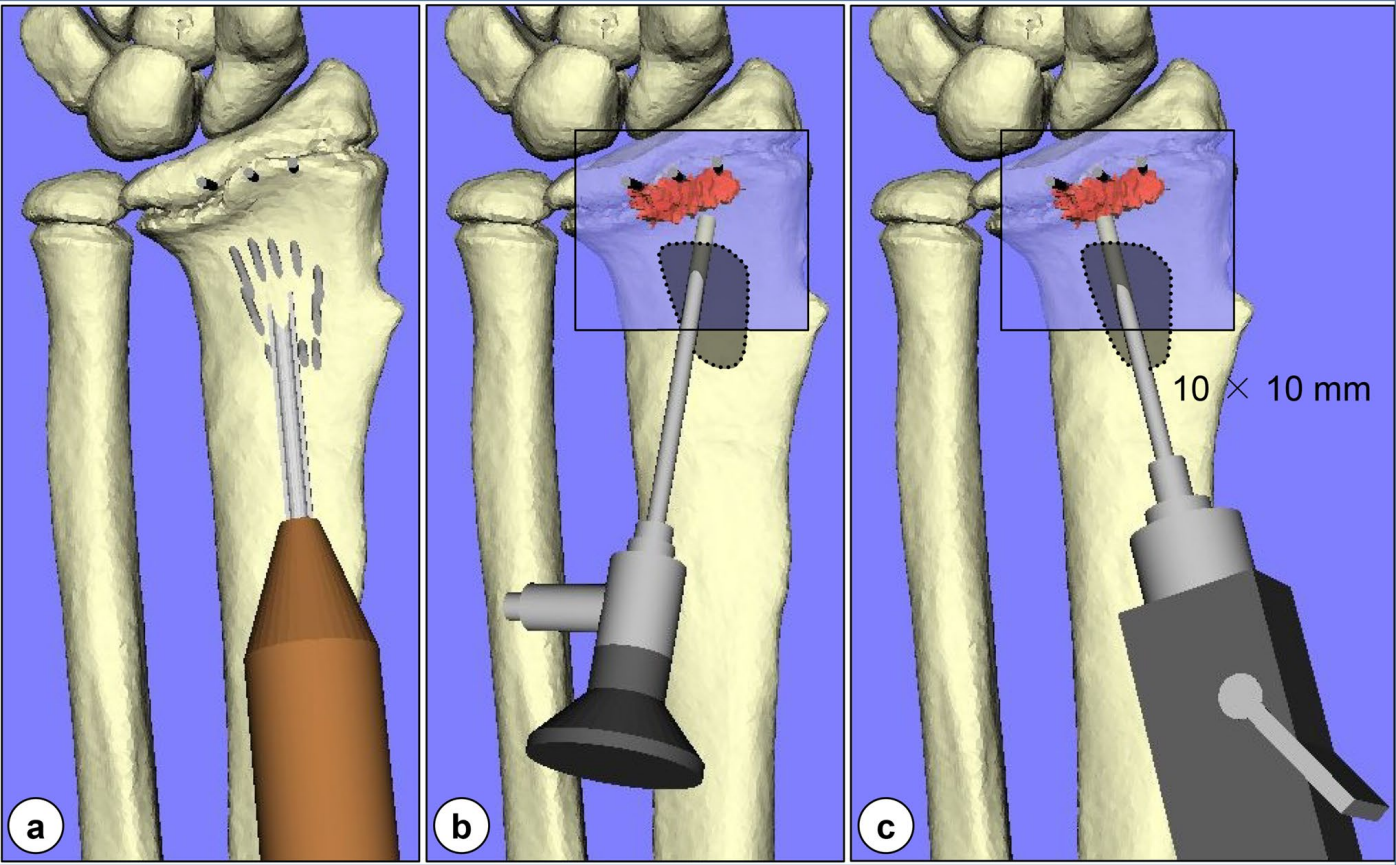
Surgical process with intramedullary endoscopic assistance: **a** creation of an osseous window of 10 × 10 mm enclosed by the wires using an osteotome; **b** direct visualization of the physeal bar using a 1.7-mm endoscope through the window; and **c** debridement of the residual bar inside the epiphysis with a motorized shaver

**Fig. 5:**
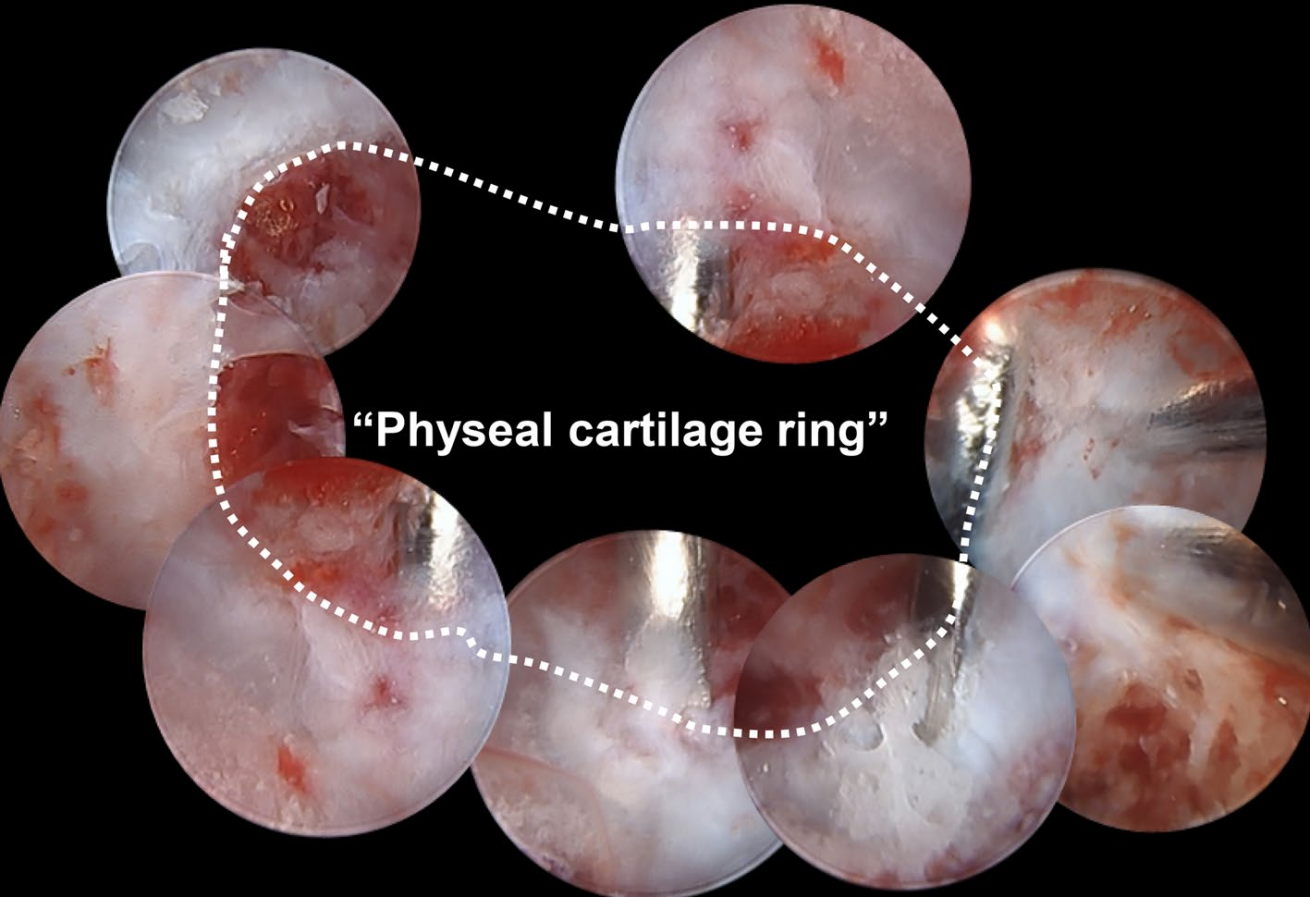
The “physeal cartilage ring” nearly 360° around the border of the bar

Patients were placed in the supine position with the operative limb, with tourniquet, placed on an arm board, using the modified Henry (radio-palmar) approach. Retracting the flexor tendons to the ulnar side, the pronator quadratus muscle on the radial side was elevated by subperiosteal dissection to expose the palmar surface of the distal radius. The patient-specific guide was fitted onto the palmar surface under the fluoroscopic guidance of a K-wire designed to indicate the radial styloid tip as a reference, allowing the confirmation of the precise guide position. Next, multiple pinning with 1.0-mm K-wires was performed through the holes created on the patient-specific guide. The insertion depth of these K-wires was determined under fluoroscopic guidance using block pins designed to avoid penetration into the radiocarpal joint as points of reference. After that, the patient-specific guide was removed leaving the K-wires, which were cut at the bone surface (Fig. 3a–d). An osseous window of approximately 10 mm × 10 mm enclosed by the wires was created using an osteotome through which resection of the physeal bar was performed by advancing curettes under fluoroscopic guidance until the block pins were encountered. Finally, a 1.7-mm endoscope was used under saline-solution perfusion to verify the thoroughness of the bar excision for direct visualization (intramedullary endoscopically assisted technique). Once the normal physeal cartilage was identified, the residual bar inside the epiphysis was debrided carefully with a motorized shaver until a complete ring of the physeal cartilage was identified by alternately introducing and removing the scope and shaver (Fig. 4a–c). After nearly 360° of the “physeal cartilage ring” was observed around the border of the bar (Fig. 5) [13], some interposition material, such as autogenous body fat or surgical bone wax, was packed into the resection cavity and secured with sutures through the surrounding soft tissues as necessary.

Postoperatively, a short arm cast was applied for approximately 2 weeks. After cast removal, the range of motion exercise was gradually started.

## Case report

**Fig. 6:**
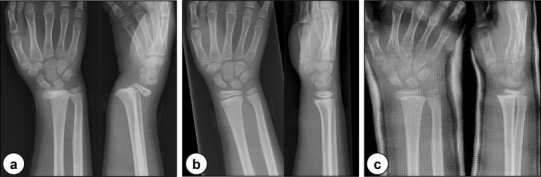
Anteroposterior (left) and lateral (right) radiographs in the initial treatment: **a** growth plate fracture of the right distal radius; **b** after closed reduction; and **c** displacement of the fracture site during cast immobilization

**Fig. 7:**
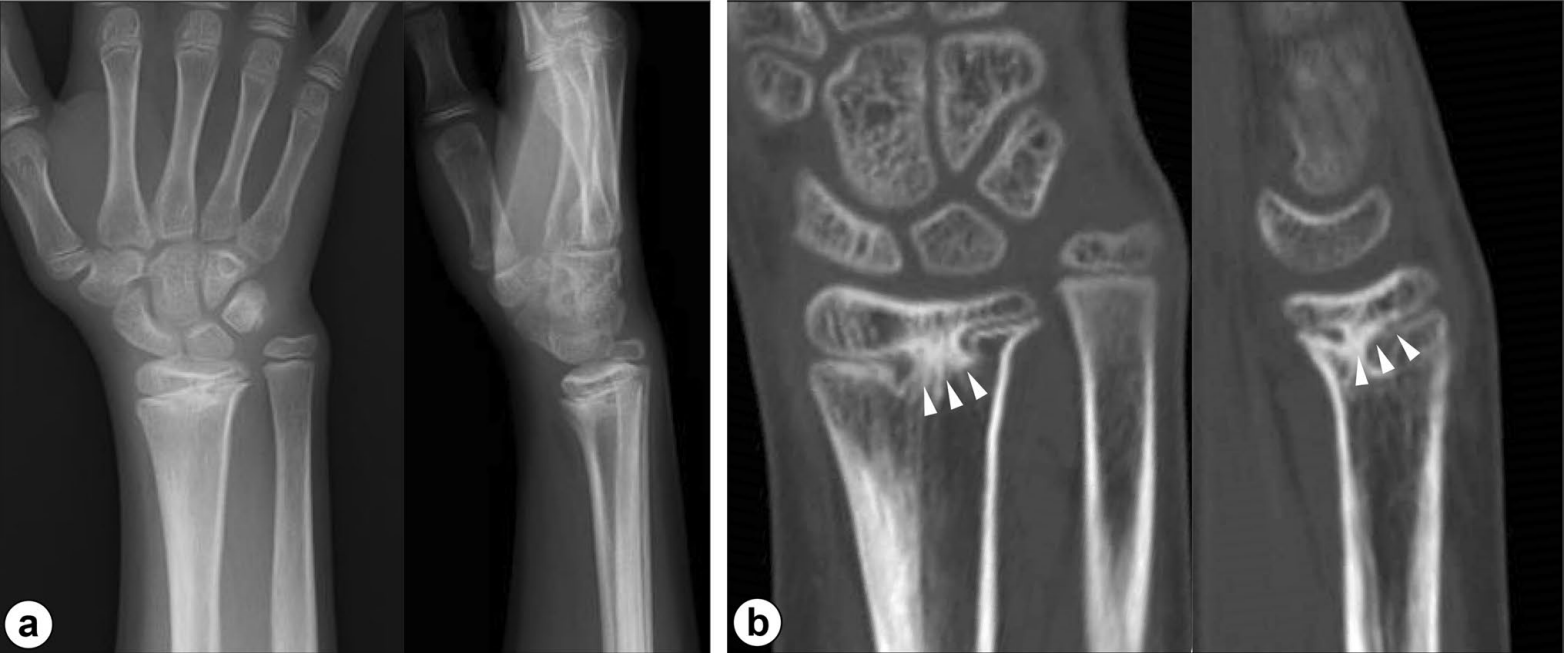
Image findings a year after injury: **a** anteroposterior (left) and lateral (right) radiographs; and **b** coronal (left) and sagittal (right) views of the CT scans showing the physeal bar (arrowhead) at the center of the growth plate

A 9-year-old girl sustained a growth plate fracture of the right distal radius (Salter–Harris type II). She was initially treated with cast immobilization following a closed reduction (Fig. 6a–c). Although the fracture healed after several weeks, wrist pain and unsightly appearance of the wrist developed within a year, following which she was referred for management. Physical examination revealed a visible radial deviation deformity of the wrist and prominence of the ulnar head with a complaint of ulnar-sided wrist pain although forearm rotation and the range of wrist flexion–extension were not impaired. Radiographs revealed a shortening deformity of the distal radius with an abnormal radial inclination of the articular surface (almost 0°; Fig. 7a), and CT revealed that the physeal bar existed at the center of the growth plate (Fig. 7b). CT scan was performed on both wrists to evaluate the deformity and for preoperative simulation with a low-radiation setting (scan pitch, 0.562:1; speed, 5.62 mm/rot, 30 mA, 120 kV) [14]. On the basis of these findings, she was diagnosed with partial physeal arrest, with significant growth remaining. A two-stage operation was planned to remove the physeal bar with the Langenskiöld procedure after gradual lengthening with distraction osteogenesis. The first stage aimed to correct the deformities of the wrist, and the second stage was intended to reestablish the physeal growth. Informed consent was obtained from the patient’s guardians to report the procedure. All procedures were approved by the Ethics Committee of the institution (registration number, 13558), and adhered to all of the recommended guidelines of the institution for an experimental investigation involving human subjects.

### Gradual lengthening with distraction osteogenesis

**Fig. 8:**
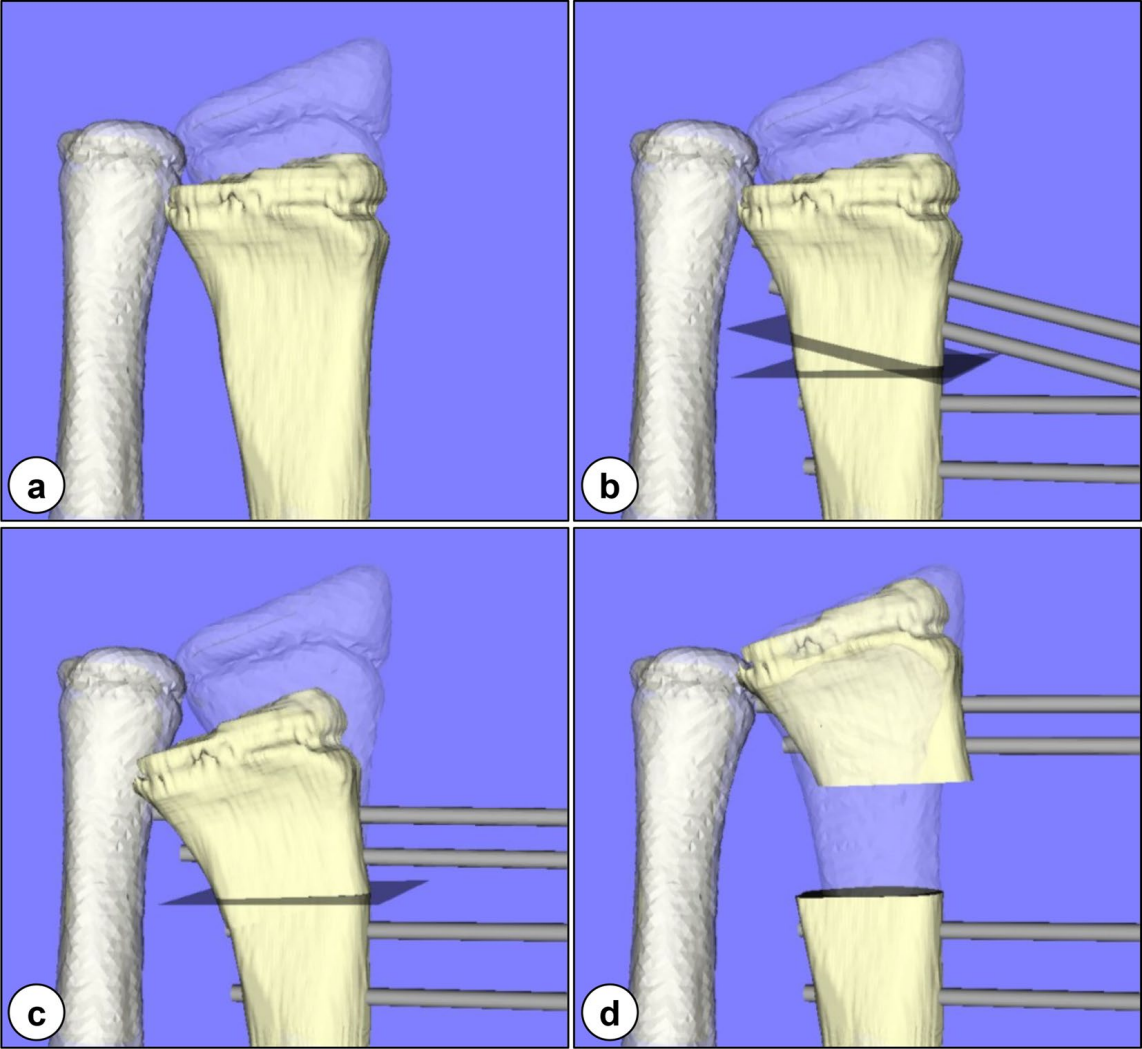
Preoperative planning: **a** distal part of the deformed radius (beige) superimposed onto the normal mirror image of the counterpart (transparent white); **b** spatial position of the planes for osteotomy and the screws for the external fixator; **c** corrected radius at the beginning of lengthening; and **d** at the end of lengthening by 25 mm

**Fig. 9:**
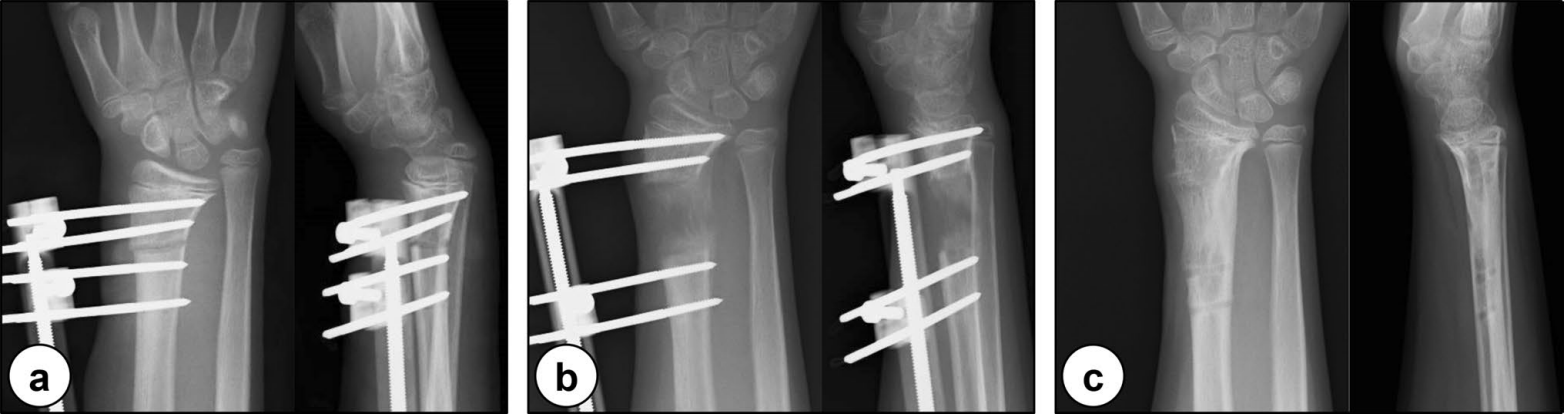
Anteroposterior (left) and lateral (right) radiographs during the gradual lengthening with distraction osteogenesis: **a** at the start of lengthening 1 week postoperatively; **b** at the end of lengthening, 7 weeks postoperatively; and **c** at the adequate maturation of the callus at the bone interval, 18 weeks postoperatively

In the first operation, closed-wedge osteotomy was used to correct the distal radius, and lengthening was started using an external fixator (Orthofix MiniRail Fixator™; Orthofix Inc., Verona, Italy). Before the operation, 3-D computer models of the bilateral radius and ulna were created from the CT data. Referring to the standard mirror image of the contralateral counterparts, the spatial position of the planes for osteotomy was estimated. Additionally, precise information on screw positions and directions for the external fixator was gained followed by computational lengthening of the distal fragment of the radius by 25 mm (Fig. 8). The operation was performed according to this preoperative planning and the osteotomy site was lengthened from 1 week postoperatively to 7 weeks (total lengthening, 25 mm; as planned). The external fixator was removed 18 weeks postoperatively when adequate maturation of the callus at the bone interval and complete correction of the deformities were confirmed through plain radiographs (Fig. 9a–c).

### PBR (Langenskiöld procedure)

**Fig. 10:**
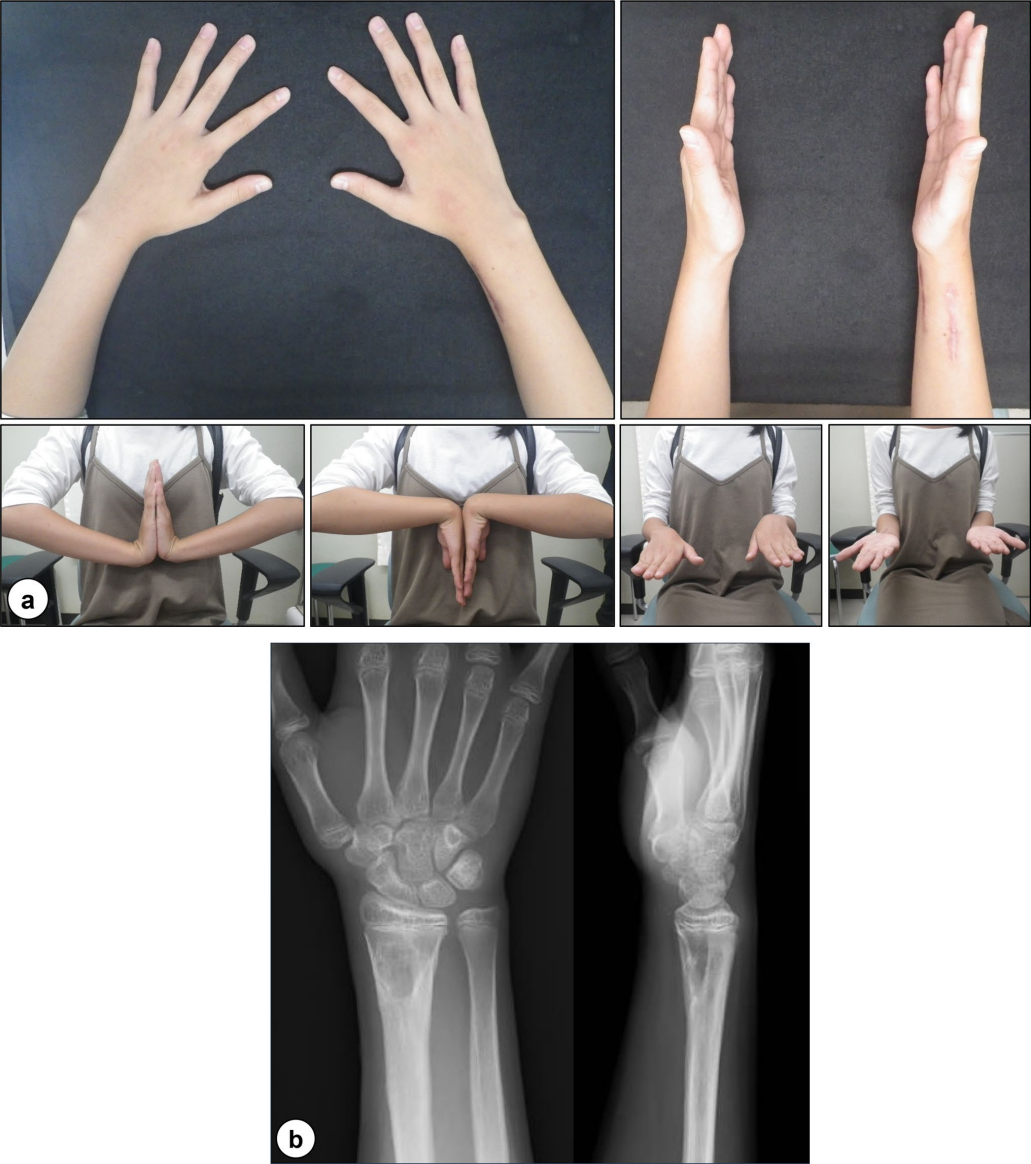
Postoperative radiographs: **a** photographs and **b** anteroposterior (left) and lateral (right) radiographs

To plan the second operation, the affected radius was rescanned on CT after lengthening was complete. The scan revealed that the area of the osseous bar was approximately 20% of the growth plate. At this time, the patient was 11 years old with the predicted growth of approximately 25 mm in the distal ulna [15]. The 3-D computer models of the radius and the physeal bar were created using these data. In addition, the patient-specific guide was designed as a surgical guide to target the physeal bar model surrounded by multiple K-wires. PBR was performed using this patient-specific guide as described previously, and with careful debridement with a motorized shaver, confirmed the thoroughness of the bar excision for direct visualization using the 1.7-mm endoscope once the “physeal cartilage ring” was observed [13]. After that, the surgical bone wax was interposed into the space. Postoperatively, the affected limb remained immobilized for 2 weeks. After 2 years of PBR, no recurrence of the wrist deformities was observed, and the patient did not complain of any pain and restriction of the motion. Furthermore, radiographs demonstrated no growth disturbance and restoration of normal length of forearm bones, which remained constant since the first operation (Fig. 10a, b). The patient regained full activity of her wrist and was able to participate in athletics.

## Discussion

The Langenskiöld procedure, known for the management of the partial physeal arrest, facilitates the reestablishment of the physeal growth in the extremity [11]. This procedure aimed to remove the fibrous or bony bar and place interposition material into the resection cavity to permit the remaining growth [3]. Usually, this procedure is indicated when less than 50% of the physis is damaged and more than 2 years of the growth remains in the affected growth plate [10, 12, 16]. As for the interposition material, the surgical bone wax is often preferred. It has the advantage of avoiding an accessory incision especially for a young girl, although it is not reabsorbed once placed. In the present case, the ulna was still growing and the recurrence of the wrist deformity was anticipated; therefore, it was considered that the same clinical outcome would not have expected, if PBR had not been performed. To date, several studies have reported the clinical outcome of this technique, and their success rate was relatively high; however, a majority of them were for lower extremities and cases for forearm bones remain limited [5, 7, 11, 13, 16–18]. Thus, few data are available on the technical tricks of this procedure in the forearm bones with the aim of precise removal of the physeal bar.

In the present case, the patient-specific guide was combined with endoscopy to achieve complete removal of the bar. The advantage of this procedure is the potential to provide precise removal of the physeal bar. The surgical instrument designed specifically for the patient was applied, simplifying the surgical procedures and allowing accurate reproduction of the preoperative 3-D simulation. Studies have described the use of the guide as a promising technique. They have demonstrated good clinical outcomes [19–25] and high accuracy [26–28] in performing corrective osteotomies for upper limb deformities, and have reported its utility for the intra-articular correction of the distal radius [29, 30]. The 1.0-mm expansion of the physeal bar was defined as a target knowing that approximately 0.5 mm is the mean error in creating the models [14], and the diameter of the K-wires mentioned later is 0.5 mm. The segmented 3-D reconstruction model of the physeal bar not only facilitated spatial identification of the size and location of the bar but also suggested the removal without inadequacy and excess. Another advantage of this procedure is the ability to confirm the complete removal of the bar and conservation of healthy physeal cartilage using endoscopy. Its direct visualization enabled intraoperative confirmation of the residual physeal bar, which would have been difficult to identify only with fluoroscopic guidance. Using an endoscope and shaver by alternately inserting them through the osseous window was an effective technique for accurate resection of the bar. After confirming the completion of the ring of physis, “physeal cartilage ring,” around the endoscopic field of view, the precise takedown was confirmed [13].

Nonetheless, this method has several limitations. The creation of bone models and simulation of the operation are time-consuming activities, not to mention approximately 2 weeks were required to manufacture the instruments. Besides, the total cost of this procedure ranges approximately €500–950 ($625–1200 USD) per case, rendering it an expensive procedure. In this case, the physes of the radius and ulna were not yet completely closed, even though 2 years had passed after PBR. Thus, careful observation is imperative until bone growth is complete. Multiple pinning or bar excision poses a potential risk of damaging the articular cartilage, despite the use of block pins designed to avoid penetration under fluoroscopic guidance. By evaluating the insertion depth with drill bits of a defined length rather than K-wires (drill bit technique), the surgeons would have more precisely specified the depth and reduced the fluoroscopic time. The “monorail” type external fixator was used in the gradual lengthening surgery, wherein the distal fragments are lengthened only in the straightforward direction. Despite preoperatively simulating the amount of correction of the articular surface, the lengthening is not always performed as planned. In such cases, multidirectional adjustment in the lengthening is occasionally required during the course. Finally, this method requires multiple CT scans if staged surgery is planned, as in this case, and high radiation exposure could be a matter of concern for children; however, our study protocol of the CT condition reduced the radiation exposure to less than one-tenth of the normal condition [14]. Despite these shortcomings, this method could provide a useful surgical option for the partial physeal arrest of the distal radius. Nevertheless, further confirmatory studies with larger number of patients are warranted to validate the reliability of this modified technique.
